# *Fusobacterium nucleatum*: The Opportunistic Pathogen of Periodontal and Peri-Implant Diseases

**DOI:** 10.3389/fmicb.2022.860149

**Published:** 2022-03-11

**Authors:** Yanchi Chen, Tao Shi, Yiling Li, Linyang Huang, Derong Yin

**Affiliations:** State Key Laboratory of Oral Diseases, National Clinical Research Center for Oral Diseases, West China Hospital of Stomatology, Sichuan University, Chengdu, China

**Keywords:** *Fusobacterium nucleatum*, periodontal diseases, peri-implant diseases, oral microbiology, pathogeny

## Abstract

Peri-implant diseases are considered to be a chronic destructive inflammatory destruction/damage occurring in soft and hard peri-implant tissues during the patient’s perennial use after implant restoration and have attracted much attention because of their high incidence. Although most studies seem to suggest that the pathogenesis of peri-implant diseases is similar to that of periodontal diseases and that both begin with microbial infection, the specific mechanism of peri-implant diseases remains unclear. As an oral opportunistic pathogen, *Fusobacterium nucleatum* (*F. nucleatum*) has been demonstrated to be vital for the occurrence and development of many oral infectious diseases, especially periodontal diseases. More notably, the latest relevant studies suggest that *F. nucleatum* may contribute to the occurrence and development of peri-implant diseases. Considering the close connection between peri-implant diseases and periodontal diseases, a summary of the role of *Fusobacterium nucleatum* in periodontal diseases may provide more research directions and ideas for the peri-implantation mechanism. In this review, we summarize the effects of *F. nucleatum* on periodontal diseases by biofilm formation, host infection, and host response, and then we establish the relationship between periodontal and peri-implant diseases. Based on the above aspects, we discuss the importance and potential value of *F. nucleatum* in peri-implant diseases.

## Introduction

Peri-implant diseases, including peri-implant mucositis and peri-implantitis, occur in the soft and hard peri-implant tissues around implants during long-term usage after implant restoration (Albrektsson et al., 2016; Papathanasiou et al., 2016; Schwarz et al., 2018). Peri-implant mucositis is mucosal inflammation in peri-implant soft tissue and is clinically characterized by bleeding on gentle probing without supporting bone loss (Berglundh et al., 2018a). In contrast, peri-implantitis is a more serious pathological condition involving the implant supporting tissues, which leads to increased probing depths, bleeding on probing (BOP), and radiographic loss of marginal bone (Heitz-Mayfield and Salvi, 2018; Schwarz et al., 2018; Berglundh et al., 2018a). According to statistics, the prevalence rate of peri-implant mucositis exceeds 50%, while peri-implantitis occurs in approximately 20% of implants (Tarnow, 2016; Buser et al., 2017; Ting et al., 2018). Although several factors, such as the diagnosis method and statistical sources, may affect the results and lead to some biases, the pathogenesis and treatment strategies for peri-implant diseases are momentous issues that have recently received widespread attention but have not yet been resolved (Ritzer et al., 2017; Smith et al., 2017; Berglundh et al., 2018b).

Current studies consider that microbial biofilm accumulation may be a major cause of diseases, and therefore, a complete understanding of the peri-implant microbiota is important for treatment planning. With continuous advances in microbiological techniques, including culture-dependent methods, molecular methods, and sequencing methods, some common pathogens, such as *Tannerella forsythia*, *Porphyromonas gingivalis*, *Prevotella intermedia*, and *Fusobacterium nucleatum*, have been detected in peri-implant clinical or laboratory samples (Charalampakis and Belibasakis, 2015; Sahrmann et al., 2020). *Fusobacterium nucleatum* is a common but unique microorganism in the oral environment and has aroused much interest as it participates in not only dental but also extraoral and systemic infectious diseases (Brennan and Garrett, 2019). In the oral environment, on the one hand, *F. nucleatum* could establish a symbiotic relationship with the host under a healthy state (Dukka et al., 2021). On the other hand, *F. nucleatum* could destroy the balance as a pathogen, interacting with other pathogens and leading to severe oral diseases (Lamont et al., 2018; Stokowa-Soltys et al., 2021). Existing research has explained how *F. nucleatum* affects the initiation and development of periodontal diseases to a large extent, but few studies have focused on the relationship between peri-implant diseases and *F. nucleatum*. Since periodontal diseases and peri-implant diseases exhibit core similarities but also have specific unique characteristics, can the current findings on the association of periodontal disease with *F. nucleatum*, along with its methodology, be applied to research the pathogenesis of peri-implant diseases? In this review, we mainly focus on summarizing the essential mechanism of *F. nucleatum* in periodontal diseases and discuss the potential value of *F. nucleatum* in the pathogenesis of peri-implant diseases.

## The Role of *Fusobacterium nucleatum* in Periodontal Diseases

### Biofilm Formation

It is widely accepted that during the transition from a healthy state to an inflammatory state, the composition of dental plaque undergoes a series of complex evolutions. Initial colonizers, such as *Actinomyces* and *Streptococci*, are the first microorganisms that adhere to the tooth surface, which contribute to the subsequent coaggregation (Sanchez et al., 2011), and late colonizers, such as *P. gingivalis* and *Treponema denticola*, are mostly known as periodontal pathogens that bind to previously bound bacteria (Kolenbrander et al., 2010). In the process of biofilm maturation, however, the intermediate colonizer *F. nucleatum* acts as a joint and corner stone organism that characteristically binds to representatives of nearly all colonizers, ranging from the initial to the late colonizers (Thurnheer et al., 2019; Zhang et al., 2019).

One of the most well-known adhesins of *F. nucleatum* is RadD, which contributes to interspecies coaggregation and multispecies biofilm formation. RadD is an outer membrane protein with a high molecular weight (nearly 350 KDa) that is isolated from wild-type *F. nucleatum* and initially meditates arginine-inhibitable adhesion between *F. nucleatum* and *Streptococcus cristatus* as well as *Actinomyces naeslundii* (Edwards et al., 2007; Kaplan et al., 2009). RadD is encoded by the FN1526 gene, which is now designed as the radD gene, but the expression of RadD may be controlled by a recently revealed Fusobacteria two-component signal transduction system, CarRs (Kaplan et al., 2009; Wu et al., 2021). Using a radD mutant, Kaplan et al. (2009) observed the suppression of *F. nucleatum* and streptococci coadherence, and this finding demonstrated the unique character of RadD in *F. nucleatum* binding capacity for the first time. The same approach has been applied in other later RadD-binding related studies, such as the interaction between *F. nucleatum* RadD and *Streptococcus mutans* SpaP (Guo et al., 2017) and the RadD-associated coaggregation between *F. nucleatum* and *Candida albicans* (Wu et al., 2015), which have all emphasized the significant function of RadD in the coadherence between *F. nucleatum* and oral microbiota. RadD mediates adhesion not only to oral colonizers but also to intestinal colonizers. It binds to *Clostridioides difficile*, a well-known anaerobic intestinal pathogen (Sandhu and McBride, 2018), *via* flagella on *C. difficile*, promoting biofilm formation, accelerating *C. difficile* colonization, and ultimately leading to *C. difficile* infection and severe diarrhea (Engevik et al., 2021).

**Figure 1:**
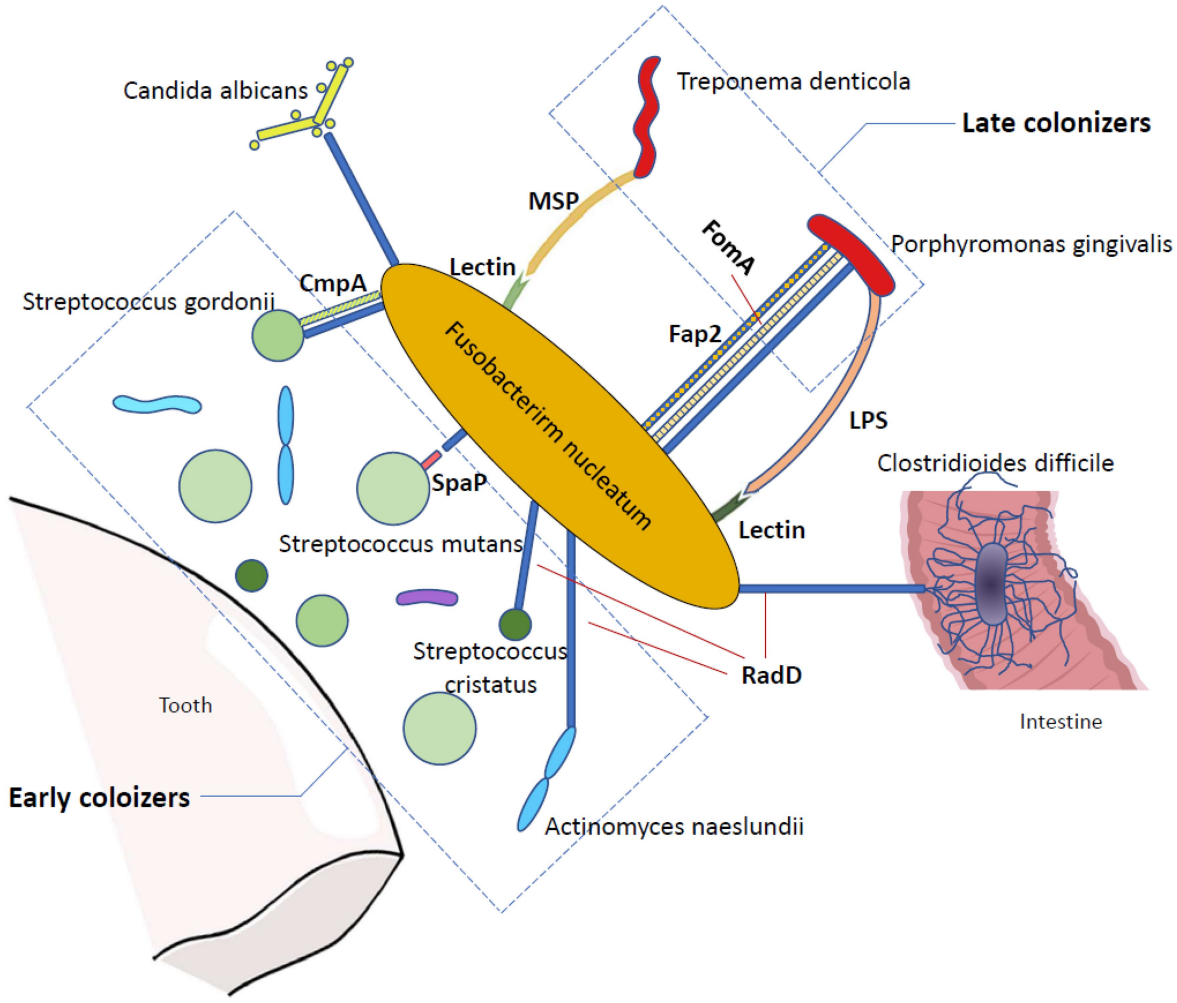
The bridging function of *Fusobacterium nucleatum* in biofilm formation. *Fusobacterium nucleatum* is a bridging organism that has a great ability to bind to many other microorganisms. In the oral cavity, *Fusobacterium nucleatum* adheres to both early and late colonizers, promoting the coaggregation of periodontal disease-related pathogens. In addition, it can bind to *Candida albicans* and *Clostridioides difficile*. RadD is one of the most profoundly studied adhesions of *Fusobacterium nucleatum* and contributes to the binding between *Fusobacterium nucleatum* and *Candida albicans*, *Streptococcus cristatus*, *Streptococcus gordonii*, *Streptococcus mutans*, *Actinomyces naeslundii*, *Porphyromonas gingivalis*, and *Clostridioides difficile via* an arginine-inhibitable method. Several other *Fusobacterium nucleatum* outer membrane proteins are also vital in bacterial coaggregation and biofilm formation, including Fap2, FomA and CmpA. Moreover, the *Fusobacterium nucleatum* outer membrane lectin also contributes to dual-species biofilm formation in a galactose-inhibitable method.

Apart from RadD, many other outer membrane proteins in *F. nucleatum* also have prominent roles in bacterial coaggregation and biofilm formation. The 42-kDa *F. nucleatum* major outer-membrane protein (FomA), which mediates coaggregation with *P. gingivalis* in periodontal pockets (Kinder and Holt, 1993), has been reported to be a crucial factor for fusobacterial biofilm-bridge function (Zhang et al., 2021). FomA cooperates with RadD and Fap2 to achieve binding between *F. nucleatum* and *P. gingivalis* (Brennan and Garrett, 2019). Another *F. nucleatum* outer membrane protein, coaggregation mediating protein (CmpA), has been targeted as it provides additional adhesion in the binding between *F. nucleatum* and *Streptococcus gordonii*, and it is equally important as RadD (Lima et al., 2017). Moreover, in addition to *F. nucleatum* outer membrane proteins, *F. nucleatum* outer membrane lectin greatly contributes to dual-species biofilm formation. Both *T. denticola* major outer sheath protein (MSP) and *P. gingivalis* lipopolysaccharide (LPS) are able to bind to the *F. nucleatum* lectin in a galactose-inhibitable manner to enhance interspecies coaggregation (Rosen and Sela, 2006; Rosen et al., 2008). Once a biofilm is formed, it can heighten the survival, invasion, and pathogenic capability of *F. nucleatum* (Gursoy et al., 2010; Horiuchi et al., 2020), which may influence the proliferation pattern of the oral epithelium and lead to early periodontal disease-related lesions (Pollanen et al., 2012; Figure 1).

### Host Infection

The important mechanism that elicits infection in the host is the remarkable adherence and/or invasive properties of *F. nucleatum*. It has been confirmed that *F. nucleatum* can bind to and/or invade a wide variety of host cells (Han et al., 2000; Dabija-Wolter et al., 2009; Fardini et al., 2011).

A specifically characterized *F. nucleatum* adhesin, Fusobacterium adhesion A (FadA), which was first found to mediate the binding between *F. nucleatum* and oral mucosal cells, has been discovered to be a crucial part of these functions (Han et al., 2005). The FadA gene is considerably conserved among two closely related oral fusobacteria, which are referred to as *F. nucleatum* and *F. periodonticum*. However, it is absent in almost all the other nonoral fusobacterial species (Han et al., 2005). Using PCR technology to detect this gene in *F. nucleatum*-positive subgingival biofilm samples, Liu et al. (2014) substantiated that the gingival index directly varies with the detection rate of FadA. The functional form of FadA, the FadA complex (FadAc), is a heterogeneous recombinant gene that is composed of pre-FadA consisting of 129 amino acid residues and mFadA consisting of 111 amino acid residues (Xu et al., 2007). Pre-FadA is a full-length peptide that processes an 18-amino acid signal peptide, which is anchored in the inner membrane, whereas mFadA exists in a mature form that is secreted from the bacterial cell outer membrane (Xu et al., 2007). Neither pre-FadA nor mFadA exhibited little virulence. The crystal structure of mFadA indicated that the mFadA monomers assemble in a head-to-tail pattern by a novel leucine chain motif (Nithianantham et al., 2009). However, this structure is a long, thin, and unstable filament that is defective in curbing the adhesion of *F. nucleatum* to host cells by itself (Xu et al., 2007; Temoin et al., 2012). When mixed with pre-FadA, the oligomers bind to each other and form short and stable heterogeneous fibrils with the help of signal peptides (Temoin et al., 2012; Meng et al., 2021). Under stress conditions, FadAc assembles into a functional amyloid-like structure, which enhances the pathogenicity of *F. nucleatum* and ultimately leads to periodontal disease and periodontal bone loss in mice, and pre-FadA is a critical component (Meng et al., 2021). In addition to adhesin, FadA also plays a critical role in invasion as it binds to cadherins, which are known as cell junction molecules (Han, 2015; Rubinstein et al., 2019). FadA binds to endothelial cell vascular endothelial cadherin, relocating its position and loosening intracellular bridges. Ultimately, the structural integrity of the endothelium is destroyed, which may enable noninvasive bacteria to spread systemically and ultimately lead to mixed infection (Fardini et al., 2011).

*Fusobacterium nucleatum* not only possesses the remarkable ability to adhere to and/or invade host cells itself but also acts as a promoter that enhances the attachment and/or internalization of some early and late colonizers. *Streptococcu*s *cristatus*, a *Streptococcu*s, is a noninvasive early colonizer that lacks a great ability to bind gingival epithelial cells and internalize by itself (Handley et al., 1991). However, noninvasive bacteria could be transported into host epithelial cells *via* the coaggregation method when coincubated with *F. nucleatum* (Edwards et al., 2006). In contrast, *P. gingivalis* is generally acknowledged as a crucial bacterium in the development of periodontitis (Reyes, 2021) that possesses invasive capacity. However, the invasive feature of *P. gingivalis* to host cells is dramatically inferior to that of *F. nucleatum* (Jang et al., 2017), which is in agreement with the finding of De Andrade et al. (2021) that *F. nucleatum* is more virulent than *P. gingivalis* to host cells. It has been demonstrated that mixed infection with *F. nucleatum* strengthens the invasion capacity of *P. gingivalis* in oral epithelial cells (Zhang et al., 2021). The “promoter” characteristic of *F. nucleatum* makes it a crucial pathogen in host mixed infection.

### Host Responses

As an opportunistic pathogen, *F. nucleatum* participates in both periodontal health and periodontal disease-related host responses. Under healthy conditions, *F. nucleatum* constantly stimulates gingival epithelial cells, which leads to the constant expression of human beta-defensin-2, an antimicrobial peptide (AMP; Krisanaprakornkit et al., 2000). AMPs are able to directly defend against invading microbes and intercommunicate with the adaptive immune system, which keeps the oral epithelia from incurring infection and contributes significantly to innate immune responses (Yin and Dale, 2007; Chung and Khanum, 2017; Magana et al., 2020). This mutual effect between *F. nucleatum* and gingival epithelial cells has been found to be most strongly mediated by *F. nucleatum*-associated β-defensin inducer (FAD-I), an *F. nucleatum* cell wall-associated protein encoded by the FN1527 gene (Gupta et al., 2010; Ghosh et al., 2018). It binds to gingival epithelial cell Toll-like receptor 1/2 (TLR-1/2) and TLR-2/6 to promote beta-defension-2 induction (Bhattacharyya et al., 2016). Several other effector molecules that also play a significant role in preserving periodontal health, including cytokines and chemokines, could be promoted by the stimulation of gingival epithelial cells by *F. nucleatum* (Krisanaprakornkit et al., 2000; Wassing et al., 2021). In the complex oral environment, these molecules are maintained under homeostasis to preserve periodontal health (Han, 2015).

**Figure 2:**
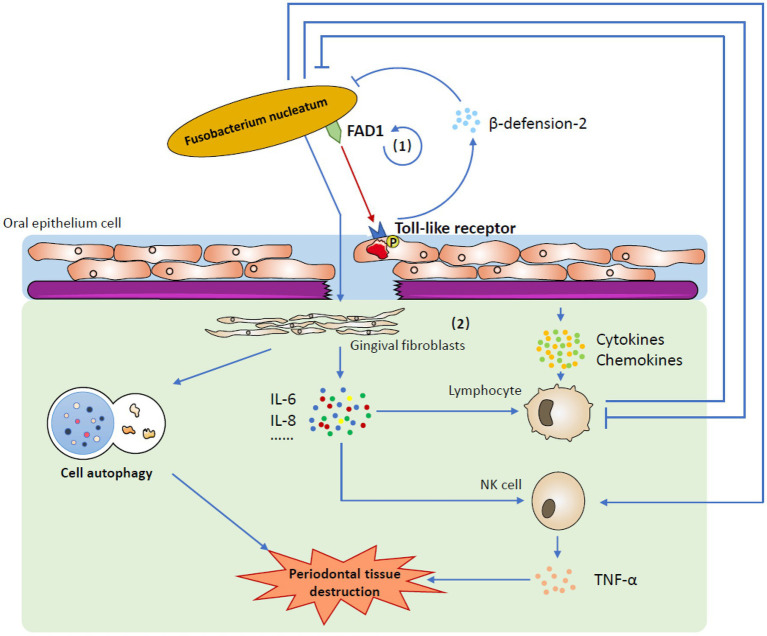
The mechanism by which *Fusobacterium nucleatum* induces host responses. (1) Under healthy conditions, one of the most important factors is that *Fusobacterium nucleatum* uses FAD-I to stimulate gingival epithelial cells to express human beta-defensin-2 *via* gingival epithelial cell Toll-like receptors. Stimulated human beta-defensin-2 helps to defend against invading microbes, including *Fusobacterium nucleatum* itself, and maintains homeostasis in the complex oral environment. (2) In a state of disease, *Fusobacterium nucleatum* destroys the epithelial barrier and affects human gingival fibroblasts. It not only induces cell autophagy but also stimulates the secretion of several cytokines and chemokines. When challenged by *Fusobacterium nucleatum*, the immune system is activated to eliminate invasive bacteria. However, *Fusobacterium nucleatum* could restrain the human immune system to reduce this removal effect. Moreover, the production of TNF-α from activated NK cells may lead to periodontal tissue destruction.

In a state of disease, the balance is broken, and *F. nucleatum* destroys the epithelial barrier, becoming a pathogen and causing malignant host responses. When the integrity of the epithelium is destroyed, human gingival fibroblasts (GFs), which are most abundant in periodontal connective tissue, become the first line of defense against *F. nucleatum* invasion (Ahn et al., 2017b; Naruishi and Nagata, 2018). On the one hand, *F. nucleatum* upregulates the intracellular ROS level, facilitating cell autophagy (Kang et al., 2019); on the other hand, it stimulates the secretion of a number of cytokines, such as IL-6 and IL-8, which are able to recruit immune cells to fight against infection (Ahn et al., 2017a). It seems that with the activation of acquired immunity, the invaded *F. nucleatum* could be eliminated very quickly. However, the immune system is closely affected by *F. nucleatum*, so the case is more complicated. One important virulence factor is that *F. nucleatum* can use its Fap2 and RadD outer membrane proteins to attack human lymphocytes, which enables *F. nucleatum* to combat the human immune system and kill lymphocytes (Kaplan et al., 2010). Moreover, the influence of *F. nucleatum* on NK cells could promote the production of TNF-α, which results in more serious alveolar bone loss in mice and probably worsens pathological outcomes in humans (Chaushu et al., 2012; Figure 2; Table 1).

**Table 1:** Virulence factors of *Fusobacterium nucleatum* in periodontal diseases.

Virulence factors			Actions
Adhesions		RadD	Interspecies coaggregation and multispecies biofilm formation Combats the human immune system and kill lymphocytes
		FomA	Mediates coaggregation with *Porphyromonas gingivalis*
		CmpA	Provides additional adhesion with *Streptococcus gordonii*
		FadA	Binds to oral mucosal cells and cadherins
Outer membrane lectin			Enhances interspecific aggregation of *Treponema denticulatum* and *Porphyromonas gingivalis*
Promoter characteristic			Enhances the attachment and/or internalization of colonizers
FAD-I			Promotes the expression of human beta-defensin-2
Fap2			Combats the human immune system and kill lymphocytes

## Special Features of Periodontal Diseases and Peri-Implant Diseases

Considering the similarity between these two diseases, the early peri-implant mucositis and the later peri-implantitis of peri-implant diseases are comparable to gingivitis and periodontitis, respectively (Schincaglia et al., 2017).

As the early stage of the disease, both peri-implant mucositis and gingivitis cause similar symptoms, such as redness, swelling, and bleeding on gentle probing (Heitz-Mayfield and Salvi, 2018). *Fusobacterium nucleatum* may contribute to this stage by recruiting neutrophils and macrophages to the oral mucosa (Johnson et al., 2018). However, compared with gingivitis, the degree of peri-implant mucositis is more severe, which results in more suppuration and bleeding sites (Meyer et al., 2017; Heitz-Mayfield and Salvi, 2018). Gualini and Berglundh (2003) demonstrated that the proportion of neutrophils and macrophages is higher around lesions from peri-implant mucositis than from gingivitis, which accounts for the more pronounced inflammatory response observed for peri-implant mucositis than gingivitis. Except for the high content of inflammatory cells and inflammatory mediators (Belibasakis, 2014), some specific microbial taxa that have nothing to do with gingivitis have been demonstrated to be positively correlated with peri-implant mucositis (Schincaglia et al., 2017). Even though some crucial differences have been found, the pathogenesis of peri-implant mucositis and gingivitis has not been found to be fundamentally different (Gurlek et al., 2017).

The clinical symptoms of peri-implantitis and periodontitis are also nearly identical (both lead to bone resorption and abscesses; Donos, 2018). However, to date, multiple factors have been found to contribute to the differences between peri-implantitis and periodontitis. From an inflammatory point of view, the extent of peri-implantitis lesions is extraordinarily larger than that of periodontitis lesions, which nearly affects the marginal bone (Dukka et al., 2021). In agreement with peri-implant mucositis and gingivitis, the proportions, numbers and densities of infiltrated neutrophils, macrophages, plasma cells and nitric oxide synthase (iNOS)-positive cells are higher in peri-implantitis than in periodontitis, which reflects the considerably larger quantity of abscesses observed in peri-implantitis clinical symptoms (Carcuac and Berglundh, 2014; Schwarz et al., 2018; Dionigi et al., 2020). From the microorganism point of view, the main distinguishing difference between these two diseases lies in the composition of biofilms, even though both are microbial infectious diseases. First, the plaque construction of peri-implantitis is simpler than that of periodontitis, and its microbial diversity is also low (Kotsakis and Olmedo, 2021). Studies have shown that the abundance of species shared between healthy implants and teeth in most patients is less than 8% (Kotsakis and Olmedo, 2021), while the abundance of species shared between peri-implantitis and periodontitis is less than 50% (Berglundh et al., 2018b). Another finding indicating the divergence between periodontal disease and peri-implantitis biofilms is that peri-implantitis microbes are resistant to beta lactam antibiotics (Leonhardt et al., 2003; Hallstrom et al., 2012), but beta lactam antibiotics show promising therapeutic effects against periodontal disease (Lopez et al., 2006). Moreover, the red complex, which is traditionally enriched in the subgingival plaque of periodontitis, is not detectable in all patients with peri-implantitis (Sanz-Martin et al., 2017). Even when associated flora are detected, the flora tend to be present in much lower numbers in peri-implantitis than in periodontitis (Sanz-Martin et al., 2017). In addition, *Eubacterium nodatum*, *Eubacterium saphenum*, and *Slackia exigua*, for instance, can be detected near peri-implantitis, while these species are rarely detected in periodontal disease (Tamura et al., 2013).

The fundamental reason why peri-implant diseases differ from periodontal diseases in many ways is probably because there have been some discrepancies in their histological structures, especially the surface characteristics, between these two diseases. One of the most important fundamental differences is that the implant surface is directly connected with the crestal bone without involving the periodontal ligament or cementum in periodontal tissue (Ivanovski and Lee, 2018). Thus, in periodontal diseases, the initial substrate for bacterial colonization is mineralized organic dental tissue, whereas bacteria colonize the unmediated titanium base in peri-implant diseases (Kotsakis and Olmedo, 2021). These two substrates differ in chemical composition, roughness and free energy, which seriously affect subsequent microbial adhesion and biofilm formation (Teughels et al., 2006; Busscher et al., 2010; Kotsakis and Olmedo, 2021). Since different microbial taxa are assembled, it is easy to comprehend the variation in the inflammatory response between peri-implant diseases and periodontal diseases. Discrepancies in transmucosal soft tissue could also lead to a distinct inflammatory response. Moon et al. (1999) indicated that the peri-implant transmucosal connective tissue can be divided into two different units. Zone A is closely attached to the implant surface, which possesses almost no blood vessels but a large number of fibroblasts. Zone B is continuous to zone A. It contains more collagen fibers and vascular structures but fewer fibroblasts. Due to the absence of the periodontal ligament, the vascular supply of the peri-implant mucosa is derived only from the supraperiosteal blood vessels lateral to the alveolar bone crest. However, in addition to the supraperiosteal blood vessels, the vascular supply of the periodontium also comes from the periodontal ligament vascular plexus (Ivanovski and Lee, 2018). In general, the peri-implant transmucosal soft tissue contained more collagen fibers with a converted direction (Berglundh et al., 1991) and significantly fewer fibroblasts and vascular structures than periodontal soft tissue. The parallel direction of collagen fibers in peri-implant mucosa may fail to restrict inflammatory cells to the epithelium as the vertical ones do, which leads to the population of inflammatory cells among peri-implant connective tissue (Yuan et al., 2021). A lack of vascular supply has been speculated to weaken the defense capacity of peri-implant tissue and may make it more likely to be invaded by microorganisms (Ivanovski and Lee, 2018). These are all probable reasons why the extent of peri-implantitis lesions is extraordinarily larger than that of periodontitis lesions with more suppuration.

To conclude, biofilm composition and inflammatory responses are two detectable differences between periodontal diseases and peri-implant diseases that were originally related to histological structures. The surface characteristics determine the group of adhered biological species. The biofilm composition cooperates with the histological structure to influence the inflammatory response (Table 2).

**Table 2:** Differences between periodontal diseases and peri-implant diseases.

		Natural Teeth	Implants
Inflammatory response	The degree of inflammatory response	Relatively weaker	More severe Nearly affects the marginal bone
	The proportions, numbers and densities of infiltrated inflammatory cells	Lower	Higher
	Infiltration of inflammatory cells	Restricted to the epithelium	Populated among connective tissue
Biofilm composition	Plaque structure	More complex	Simpler
	Beta lactam antibiotics resistance	Positive	Negative
	Red complex	Detectable in most periodontal diseases	Lower detection rate
Histological structure	Initial substrate for bacterial colonization	Mineralized organic dental tissue	Unmediated titanium base
	Participation of periodontal ligament or cementum	Yes	No
	Vascular supply of connective tissue	Supraperiosteal blood vessels Periodontal ligament vascular plexus	Supraperiosteal blood vessels
	Collagen fibers	Amount: less Direction: vertical	Amount: more Direction: parallel

## Peri-Implant Diseases and *Fusobacterium nucleatum*

In essence, both periodontal diseases and peri-implant diseases are mixed inflammations that are microbially derived, with dental plaque being a critical factor for initiating the diseases (Kinane et al., 2017; Salvi et al., 2017). Based on this concept, several assessment methods were applied to determine the microbial profile with the aim of uncovering the pathogenesis of peri-implant diseases. Some early studies utilizing bacterial culture techniques found many microbial colonies containing Gram-negative anaerobes and motile rods around the lesion site of failed implants (Bower et al., 1989; Leonhardt et al., 1999; Mombelli and Decaillet, 2011; Charalampakis and Belibasakis, 2015). Further studies have shown that periodontal disease-associated pathogens can be detected in peri-implant diseases *via* DNA checkerboard hybridizations (Gatti et al., 2008; Maximo et al., 2009; Lee et al., 2012; Persson and Renvert, 2014). Coupled with the fact that peri-implantitis and periodontitis have very similar clinical symptoms (Donos, 2018), it seems that peri-implant diseases and periodontal diseases are two very similar bacterial infectious diseases. However, with the advancement of research methods, several distinctive lines of evidence have been suggested that demonstrate the differences between these two diseases, as mentioned above. Nonetheless, *F. nucleatum*, which has been fully studied for 20 years regarding the pathogenesis of periodontal diseases, has been recently found to be the most abundant species and comprises part of the shared core microbiome of periodontal diseases and peri-implant diseases (Belibasakis and Manoil, 2021). Although there have been many studies using *F. nucleatum* as an experimental species to investigate antibacterial or material antibacterial subjects (Tonon et al., 2021; Wei et al., 2021), the reason why *F. nucleatum* is selected seems to be ambiguous.

Similarities between periodontal diseases and peri-implant diseases could guide us to pay attention to the relationship between *F. nucleatum*, periodontal diseases and peri-implant diseases. Thus, the similarities may provide us with new ideas on the pathogenesis of *F. nucleatum* in peri-implant diseases that originate from periodontal diseases. However, the existing discrepancies oblige us to revise the comprehensively studied and established theories regarding periodontal diseases. Regarding peri-implant diseases, *F. nucleatum* may also play a role in biofilm formation, host infection and host response. However, the exact mechanism by which *F. nucleatum* affects the genesis and progression of peri-implant diseases is almost specific and must be precisely deliberated. The development of biofilms has been found to be the fundamental cause of many infectious diseases (Arciola et al., 2018; Del Pozo, 2018), including periodontal and peri-implant diseases. We have learned about the bridge characteristic of *F. nucleatum* in periodontal biofilm formation, with RadD, Fap2, FomA and CmpA mediating interspecies coaggregation. However, owing to the differences in biofilm composition between implants and teeth, how *F. nucleatum* interacts with peri-implant microbes and which outer membrane protein plays a key role may vary.

As a whole species, *F. nucleatum* is considered a core species that has the same proportion in health and disease, but it increases by a 3-log higher load during periodontal disease (Curtis et al., 2020) as the whole microbial community increases in biomass. However, it is worth noting that recent research reports that *F. nucleatum* is one of the first massively increased species during the progression of mucositis to peri-implantitis (Ghensi et al., 2020). The large increase in *F. nucleatum* in peri-implant diseases may alter the oral microenvironment differently and ultimately affect biofilm constitution (Renvert et al., 2018). Thus, the bridging characteristics of *F. nucleatum* in peri-implant diseases seem to be more distinct. The situation is more complex for subspecies. The *F. nucleatum* subspecies *vincentii* has been found to be the most abundant subspecies of *F. nucleatum* in peri-implantitis submucosa, periodontal pocket, healthy peri-implant submucosa, and healthy subgingival sulcus samples, which seems to have nothing to do with either periodontal diseases or peri-implant diseases (Yu et al., 2019; Curtis et al., 2020). However, using the 16S rRNA sequencing method, Schincaglia et al. (2017) found a distinct increase in the *F. nucleatum* subspecies *polymorphum* in the development of periodontal inflammation, which is opposite to the *F. nucleatum* observed in periodontal diseases. Rather, a newly targeted subspecies referred to as *F. nucleatum* Cluster 5 has been demonstrated to be extremely relevant to peri-implant inflammation (Ghensi et al., 2020). The more complicated discrepancies in *F. nucleatum* subspecies may also reflect a more intricate pathogenesis of *F. nucleatum* in peri-implant diseases.

**Figure 3:**
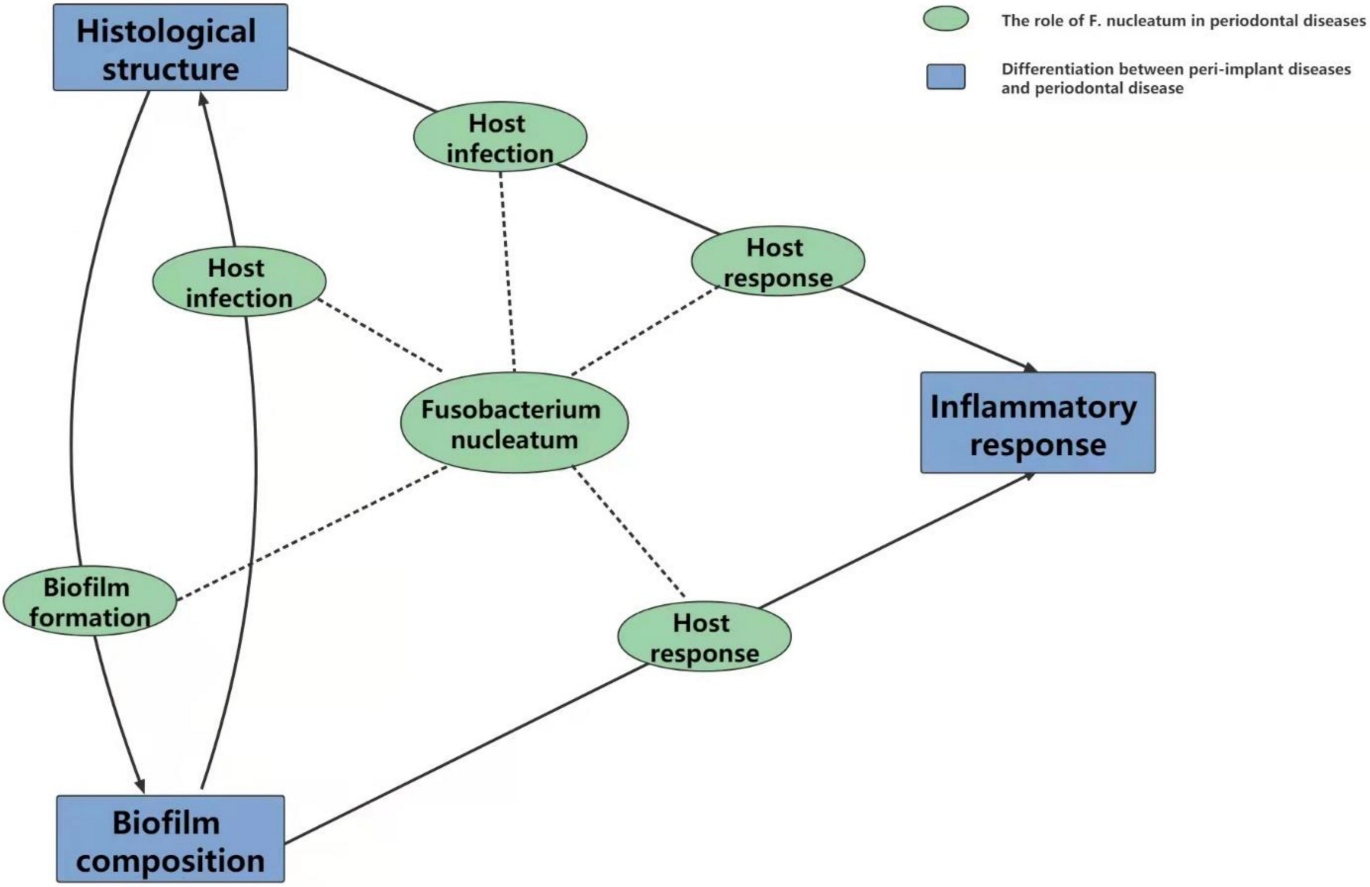
The relationship between *Fusobacterium nucleatum* periodontal diseases and peri-implant diseases. Biofilms are universally accepted as mutual and critical factors in the initiation of periodontal and peri-implant diseases. The varied substrate for bacterial colonization and probably elevated function of *Fusobacterium nucleatum* in biofilm formation may be responsible for the reported discrepancies in its composition. With the succession of dental plaque, on the one hand, *Fusobacterium nucleatum* adheres to and/or invades host cells to induce mixed infection; on the other hand, invaded *Fusobacterium nucleatum* activates the human immune system to secrete inflammatory factors and accumulate inflammatory cells. These two effects cooperate with each other to elicit the ultimate inflammatory response. Detected differences between periodontal diseases and peri-implant diseases in histological structure and inflammatory response might indicate the specific pathogenesis of *Fusobacterium nucleatum* in these two diseases individually between the host infection and host response.

Once biofilms are formed, most colonizers have the capacity to bind to and/or invade host cells. FadA is one of the most important adhesions that helps to adhere biofilms to oral mucosal cells and destroy cell–cell junctions. However, because of the converted histological structure, the method by which *F. nucleatum* elicits host infection is also probably different. The situation is more complicated for host responses. As mentioned above, both the histological structure and biofilm composition could influence the inflammatory response level. Host responses are not only related to the stimulation of the immune system by *F. nucleatum* alone but are also caused by the reciprocity between *F. nucleatum* and the surrounding substrate, and this is referred to as the “host infection” character. Owing to the divergences in biofilm composition, histological structure and *F. nucleatum*-mediated host response, inflammation is more pronounced in peri-implant diseases than in periodontal diseases. Therefore, although *F. nucleatum* can be detected in both diseases, it is highly probable that the role it plays in the progression of peri-implant disease is different from that in periodontal disease. Unfortunately, few research studies have covered these aspects (Figure 3).

## Discussion

As an oral opportunistic pathogen, *F. nucleatum* is one of the most important microorganisms in the pathogenesis of oral diseases. We have concluded how *F. nucleatum* affects the occurrence and development of periodontal diseases. In periodontitis, *F. nucleatum* first acts as a joint organism to bind to most colonizers throughout the whole process. Then, the bacteria adhere to and/or invade host cells and act as promoters, enhancing the attachment and/or internalization of some early and late colonizers. Once the balance between *F. nucleatum* and gingival epithelial cells is damaged, *F. nucleatum* destroys the epithelial barrier, becoming a pathogen and causing malignant host responses. However, in peri-implant diseases, no definite mechanism is currently available. The results of next-generation sequencing have shown that *F. nucleatum* contributes significantly to the formation and maturation of biofilms in peri-implant diseases. This indicates that *F. nucleatum* also plays a significant role in peri-implant diseases, similar to periodontal diseases. Although the bridging role of *F. nucleatum* in dental plaque is common, there are many differences between these two diseases in bacterial colonization, adhesion of microorganisms, and the formation of biofilms, leading to the high recurrence rate of peri-implantitis. Therefore, the specific mechanism by which *F. nucleatum* influences the disease seems different.

We are trying to put forward the following three aspects for exploring the pathogenesis of peri-implant diseases from the *F. nucleatum* point of view. First, more analyses and comparisons of clinical samples (healthy gingiva, healthy peri-implant, gingivitis, peri-implant mucositis, periodontitis, and peri-implantitis) are needed (de Waal et al., 2017; Yeh et al., 2019) since there have been slightly different results from different studies. Some previous studies have raised the bias that the prevalence and levels of *F. nucleatum* are not associated with peri-implantitis (Zhuang et al., 2016). *Fusobacterium nucleatum* should be confirmed as a pathogen of peri-implant diseases based on further new studies. Moreover, *F. nucleatum* subspecies should also be evaluated due to their more complicated situation. Although microbial sequencing of peri-implant diseases is available, there are currently few reports on single-cell RNA-seq and spatial transcriptome analyses around implants. If relevant data are available, it may promote the rapid progress of related research. Second, the *in vivo* dynamic changes between *F. nucleatum* and other microorganisms during the maturation of peri-implant biofilms should be considered to illustrate the biofilm formation characteristics of *F. nucleatum*. Jiang et al. (2021) demonstrated the important role of *F. nucleatum* in biofilm formation by investigating the *in vivo* microbial shift using a canine model. However, the construction of animal models of peri-implant diseases does not seem to be unified at present because in the actual process of use, the implant has an upper repair structure, which cannot be achieved with animal models, thus lacking considerations such as mechanical factors. Not only *F. nucleatum* itself but also some outer membrane proteins in *F. nucleatum*, such as RadD and FomA, should be considered. Finally, how *F. nucleatum* interacts with the host during the evolution of peri-implant diseases, including host infection and host responses, is the most operative issue that needs to be addressed. The host immune microenvironment of peri-implant issues determines the microbial composition (Wang et al., 2021). Patients with different risk levels exhibit different immune microenvironments, and *F. nucleatum* is distinctly detected in high-risk individuals (Wang et al., 2021). Host-derived marker analyses have pointed out that IL-1β is positively correlated with the probing pocket depth in experimental peri-implant models (Monje et al., 2021). High expression of IL-1β is associated with significant enrichment of *F. nucleatum* in periodontal diseases secondary to neuroinflammation (Martinez et al., 2021). The *Fusobacterium* genus tends to have a relatively higher proportion in deeper peri-implant pockets (Polymeri et al., 2021). However, whether *F. nucleatum* stimulates the expression of IL-1β, causing the typical clinical symptoms of peri-implant diseases, remains unknown. Since the relationship between *F. nucleatum* and peri-implant diseases has garnered increasing attention recently, some new technologies targeting *F. nucleatum* have been developed to treat peri-implant diseases. A new brushing solution has been tested to inhibit the development of peri-implant biofilms and kill *F. nucleatum*, which may have a wide application foreground in the treatment of peri-implant diseases (Virto et al., 2022).

In conclusion, our review summarizes the specific mechanism of *F. nucleatum* in periodontitis and analyzes the resemblances and discrepancies between periodontal diseases and peri-implant diseases. We propose a conjecture for the important role of *F. nucleatum* in periodontal diseases and provide an idea and direction for exploring the pathogenesis of peri-implant diseases. More research is still needed on how *F. nucleatum* affects the pathogenesis of peri-implant diseases.

## Author Contributions

DY and YC conceived the idea and edited the manuscript. YC and TS collected information and drafted the manuscript. YL and LH assisted in assessing the study quality and reviewing the manuscript. YC, DY, TS, YL, and LH revised the manuscript. All authors contributed to the article and approved the submitted version.

## Funding

This work was supported by Research Funding for Talent Development, West China Hospital of Stomatology, Sichuan University (RCDWJS2021-6) and Research and Development Funding, West China Hospital of Stomatology, Sichuan University (RD-02-202009).

## Conflict of Interest

The authors declare that the research was conducted in the absence of any commercial or financial relationships that could be construed as a potential conflict of interest.

## Publisher’s Note

All claims expressed in this article are solely those of the authors and do not necessarily represent those of their affiliated organizations, or those of the publisher, the editors and the reviewers. Any product that may be evaluated in this article, or claim that may be made by its manufacturer, is not guaranteed or endorsed by the publisher.
